# A digital health intervention for cardiovascular disease management in primary care (CONNECT) randomized controlled trial

**DOI:** 10.1038/s41746-020-00325-z

**Published:** 2020-09-10

**Authors:** Julie Redfern, Genevieve Coorey, John Mulley, Anish Scaria, Lis Neubeck, Nashid Hafiz, Chris Pitt, Kristie Weir, Joanna Forbes, Sharon Parker, Fiona Bampi, Alison Coenen, Gemma Enright, Annette Wong, Theresa Nguyen, Mark Harris, Nick Zwar, Clara K. Chow, Anthony Rodgers, Emma Heeley, Katie Panaretto, Annie Lau, Noel Hayman, Tim Usherwood, David Peiris

**Affiliations:** 1 Faculty of Medicine and Health, The University of Sydney, Westmead Applied Research Centre, Sydney, NSW Australia; 2 The George Institute for Global Health, UNSW, Sydney, NSW Australia; 3 Faculty of Medicine and Health, School of Public Health, University of Sydney, Sydney, NSW Australia; 4 School of Health and Social Care, Edinburgh Napier University, Edinburgh, Scotland; 5 Fiona Bampi - Cancer Australia, Australian Government, Sydney, Australia; 6 Centre for Transplant and Renal Research, Westmead Institute for Medical Research, The University of Sydney, Westmead, NSW Australia; 7 Centre for Primary Health Care and Equity, UNSW, Sydney, NSW Australia; 8 Faculty of Health Sciences & Medicine, Bond University, Gold Coast, QLD Australia; 9 School of Public Health and Community Medicine, UNSW, Sydney, Australia; 10 Centre for Chronic Disease, The University of Queensland, Brisbane, QLD Australia; 11 Australian Institute of Health Innovation, Macquarie University, Sydney, NSW Australia; 12 Queensland Health, Brisbane, QLD Australia; 13 Department of General Practice, Westmead Clinical School, Faculty of Medicine and Health, University of Sydney, Sydney, NSW Australia

**Keywords:** Preventive medicine, Ischaemia

## Abstract

Digital health applications (apps) have the potential to improve health behaviors and outcomes. We aimed to examine the effectiveness of a consumer web-based app linked to primary care electronic health records (EHRs). CONNECT was a multicenter randomized controlled trial involving patients with or at risk of cardiovascular disease (CVD) recruited from primary care (Clinical Trial registration ACTRN12613000715774). Intervention participants received an interactive app which was pre-populated and refreshed with EHR risk factor data, diagnoses and, medications. Interactive risk calculators, motivational messages and lifestyle goal tracking were also included. Control group received usual health care. Primary outcome was adherence to guideline-recommended medications (≥80% of days covered for blood pressure (BP) and statin medications). Secondary outcomes included attainment of risk factor targets and eHealth literacy. In total, 934 patients were recruited; mean age 67.6 (±8.1) years. At 12 months, the proportion with >80% days covered with recommended medicines was low overall and there was no difference between the groups (32.8% vs. 29.9%; relative risk [RR] 1.07 [95% CI, 0.88–1.20] *p* = 0.49). There was borderline improvement in the proportion meeting BP and LDL targets in intervention vs. control (17.1% vs. 12.1% RR 1.40 [95% CI, 0.97–2.03] *p* = 0.07). The intervention was associated with increased attainment of physical activity targets (87.0% intervention vs. 79.7% control, *p* = 0.02) and e-health literacy scores (72.6% intervention vs. 64.0% control, *p* = 0.02). In conclusion, a consumer app integrated with primary health care EHRs was not effective in increasing medication adherence. Borderline improvements in risk factors and modest behavior changes were observed.

## Introduction

Cardiovascular disease (CVD) is responsible for most of the global burden of non-communicable diseases (NCD) accounting for over 17 million deaths globally in 2016^1^. Internationally, guidelines place adherence to prevention medication and, healthy lifestyle behaviors at the core of CVD risk management, primary and secondary prevention recommendations^2,3^. However, use of evidence-based medications and lifestyle change are typically suboptimal^4^ and with an aging population the health burden is escalating. Thus, implementation of primary and secondary prevention strategies (such as healthy living, adherence to medicines) are an international priority requiring development and testing of innovative and scalable strategies that are evidence-based and better support patients^5^.

Major advances in internet and mobile technology over the past decade provide potential solutions to reduce the burden of CVD and broaden the reach of health care. Worldwide, more than five billion people own mobile phones^6^ and opportunities to deliver healthcare digitally are expanding exponentially with strategies such as internet portals, data-driven precision medicine and smartphone applications (apps)^7^. Although scientific evidence of their effectiveness is growing, research lags behind the rapid emergence and adoption of technology innovations targeting health-related behaviors. Benefits of interactive internet portals have been demonstrated in managing chronic conditions^8^. Our randomized controlled trial (RCT) found a physician-focused decision support tool to be effective in increasing CVD risk assessment when embedded within the primary care clinical record system^9^. In particular, personalized risk score information that is explained on a visually interesting interface, can make the impact of improving biometric risk factor values (for example, blood pressure), or behaviors (for example, smoking cessation), more compelling^9^. Hypothesized as a useful springboard to more engagement by patients with CVD risk factor control, the concept was adapted to a consumer-facing resource in the current trial. Other trials have demonstrated the benefits of apps for improving medication adherence^10^ and text messages for cardiovascular risk reduction^11^. However, to the best of our knowledge these interventions are almost all stand-alone where data is entered into the system manually and they are not integrated with the patient’s electronic health record (EHR).

Despite the potential for access to one’s EHR to increase and improve consumer engagement with disease prevention actions, relatively little is known about the effectiveness of such interventions for risk factor control. Personal EHRs now form a core component of many national health reform strategies^12^ but often stand-alone from consumer-controlled devices or apps. In the Australian primary care setting, EHRs offer software systems that assist clinicians with drug prescribing, referrals, coordination of care, clinical coding, billing, quality improvement activities and, reporting^13^. According to a recent American survey, over two-thirds of adults over 55 years of age own a smartphone and over 85% use the internet with the numbers are increasing annually^14^. As such, use of EHRs to auto-populate consumer-focused digital health interventions has promise, but robust evidence is not available about effectiveness in reducing CVD risk. Therefore, the aim of this study was to evaluate the effect of a consumer-focused digital health intervention, integrated with each participant’s primary care EHR, on guideline-recommended medication adherence, cardiovascular risk factor control and, lifestyle behaviors at one year in people at moderate to high risk of CVD.

## Results

### Overview of cohort

**Fig. 1:**
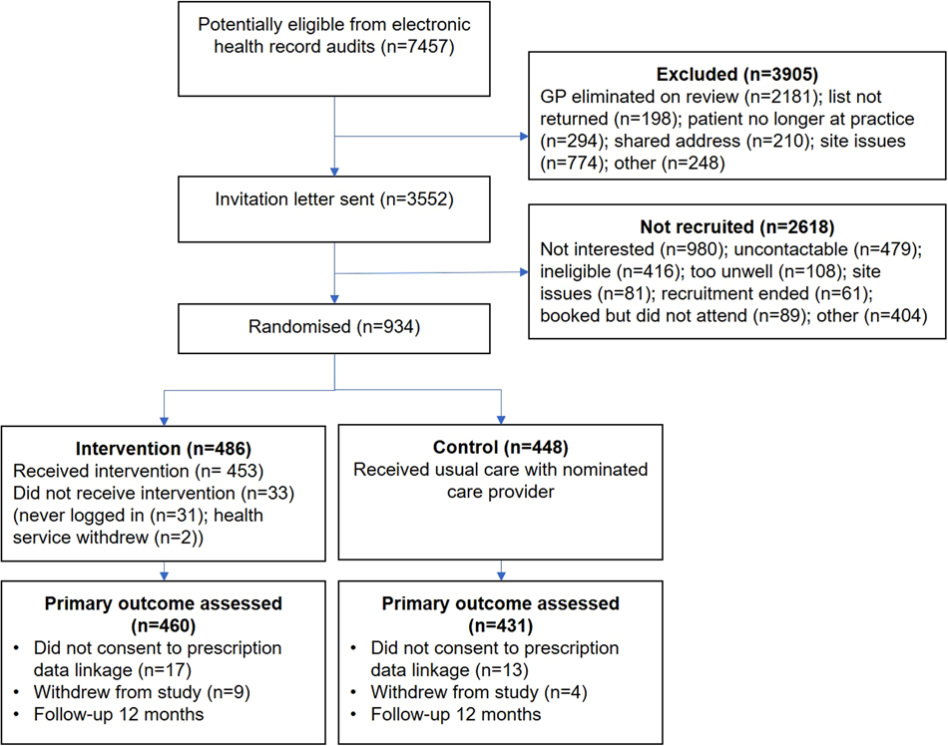
Participant flow. GP general practitioner.

In total, 7457 potentially eligible patients were identified using the primary care EHR and 3905 were excluded by their GP. We approached 3552 patients, 2618 did not meet eligibility criteria or declined participation and 934 were enrolled and randomized (Fig. 1). At 12-month follow-up 13 participants had withdrawn from the study and 30 did not consent to data linkage to access pharmacy dispensing data (Fig. 1). At baseline, the groups were well matched for demographics, cardiovascular risk factors and medication prescriptions and the mean age of participants was 67.6 (±8.1) years, 77% were male and 41% had existing CVD (Table 1). One-third of participants had existing coronary heart disease (33.3%), peripheral arterial disease (3.6%), chronic kidney disease (3.0%), atrial fibrillation (10.8%), heart failure (1.1%), and a previous stroke (9.3%).

**Table 1:** Baseline characteristics.

	Intervention^a^ (*N* = 486)	Control^a^ (*N* = 448)	Total (*N* = 934)
*Demographics*			
Age, mean (SD) years	66.8 (8.4)	68.4 (7.8)	67.6 (8.1)
Male, *n* (%)	368 (75.7)	348 (77.7)	716 (76.7)
Ethnicity, *n* (%)			
Caucasian	406 (83.5)	396 (88.4)	802 (85.9)
Asian	22 (4.5)	17 (3.8)	39 (4.2)
Aboriginal or Torres Strait Islander	27 (5.6)	10 (3.8)	37 (4.0)
Other	31 (6.4)	25 (5.6)	56 (6.0)
Education <12 years, *n* (%)	15 (3.1)	13 (2.9)	28 (3.0)
Weekly household income, *n* (%)			
$AU0–799	105 (21.7)	96 (21.4)	201 (21.6)
$AU800–1999	180 (37.1)	155 (34.6)	335 (35.9)
>$AU2000/week	116 (24.0)	119 (26.5)	235 (25.2)
No response	83 (17.1)	78 (17.4)	161 (17.3)
*Clinical data+risk factors*			
High risk of cardiovascular disease, *n* (%)	285 (58.6)	266 (59.4)	551 (59.0)
Existing cardiovascular disease, *n* (%)	201 (41.4)	182 (40.6)	383 (41.0)
Diabetes	160 (32.9)	111 (24.8)	271 (29.0)
Mean body mass index (SD) (kg/m^2^)	29.9 (5.7)	29.7 (5.1)	29.8 (5.4)
Body mass index ≥30 kg/m^2^, *N* (%)	205 (42.2)	188 (42.1)	393 (42.1)
Waist circumference, mean (SD) (cm)	105.7 (14.9)	106.4 (13.6)	106.0 (14.3)
Mean systolic blood pressure (SD) (mmHg)	137.3 (15.9)	139.0 (16.6)	138.1 (16.3)
Mean diastolic blood pressure (SD) (mmHg)	78.9 (10.6)	79.8 (10.8)	79.3 (10.7)
LDL-C, mean (SD) (mmol/L)	2.6 (1.04)	2.6 (0.98)	2.6 (1.01)
Meeting target for BP^b^, *n* (%)	195 (40.1)	165 (36.8)	360 (38.5)
LDL-C ≤ 2 mmol/L, *n*/*N* (%)	137/438 (31.3)	121/411 (29.4)	258/849 (30.4)
Meeting BP and LDL target^c^ *n*/*N* (%)	54/438 (12.3)	46/411 (11.2)	100/849 (11.8)
HbA1c, mean (SD) (mmol/mol)	7.0 (1.2)	7.1 (1.3)	7.0 (1.3)
Current smoker, *n*/*N* (%)	63/483 (13.0)	57/443 (12.9)	120/926 (13.0)
Physically inactive, *n*/*N* (%)	61/419 (14.6)	62/387 (16.0)	123/806 (15.3)
*Quality of life and health literacy*			
eHeals score, mean (SD)	27.0 (6.43)	27.0 (6.41)	27.0 (6.42)
eHEALS score ≥26, *n*/*N* (%)	326/483 (67.5)	287/448 (64.1)	613/931 (65.8)
EQ5D score/100, mean (SD)	80.1 (13.8)	79.4 (13.8)	79.8 (13.8)
*Self-reported medication use*			
Lipid lowering, *n*/*N* (%)	259/460 (56.3)	212/431 (49.2)	471/891 (52.9)
Antihypertensives, *n*/*N* (%)	287/460 (62.4)	275/431 (63.8)	562/891 (63.1)
Antithrombotics, *n*/*N* (%)	180/460 (39.1)	183/431 (42.5)	363/891 (40.7)
≥80% medication days covered, *n*/*N* (%)	133/460 (28.9)	122/431 (28.3)	255 (28.6)

*N* number of participants in denominator, *n* number of participants in the numerator, *SD* standard deviation, *LDL-C* low density lipoprotein cholesterol, *HbA1c* glycated hemoglobin, *EQ5D* EuroQual 5D.

^a^Denominators are included where the denominator differed from the column total.

^b^BP target defined as: ≤130/80 mmHg for CVD, diabetes or albuminuria or ≤140/90 mmHg for all others.

^c^LDL-cholesterol target defined as <2.0 mmol/L.

### Results for primary and secondary outcomes

**Fig. 2:**
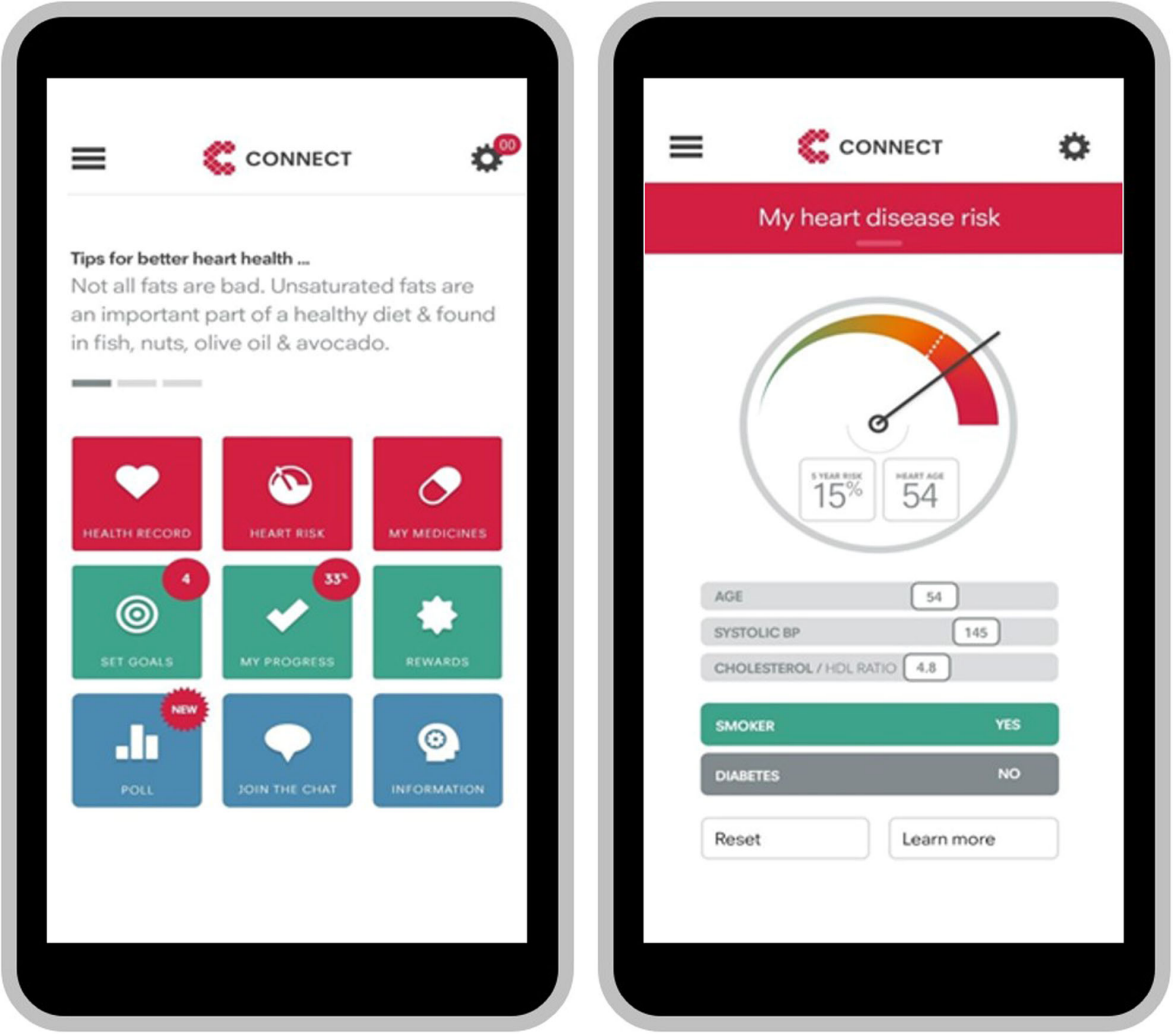
Intervention screen shots.

**Fig. 3:**
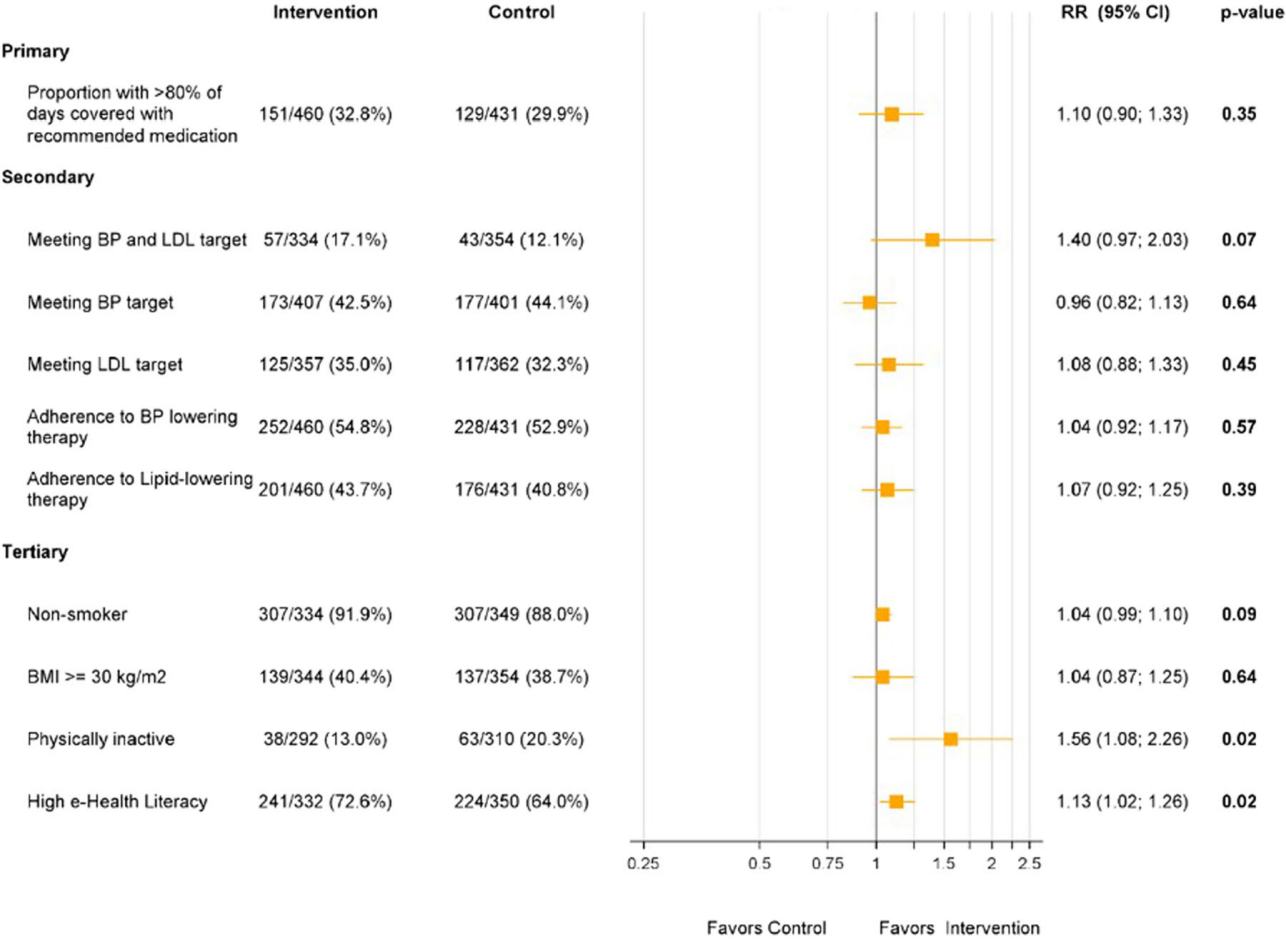
Trial outcomes. CI confidence interval, RR relative risk, BP blood pressure, LDL low-density lipoprotein cholesterol, BMI body mass index, kg kilogram, m meter.

**Fig. 4:**
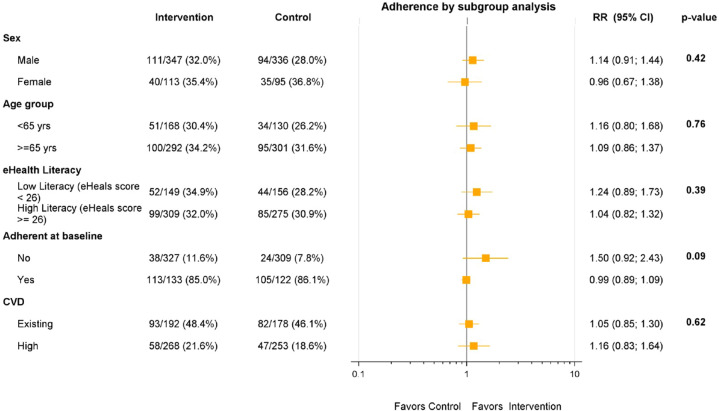
Sub-group analyses for the primary outcome. yrs years, eHEALS eHealth literacy score, CVD cardiovascular disease.

Overall, 93% (451/486) of intervention group participants commenced use of the intervention (Fig. 2). Thereafter, participants were classified as non-adopters (no logins after the training session—13%, 58/451), low-users (at least one login any across any 3 months of the follow-up period—47%, 211/451) or high-users (at least one login in any 4 months of the follow-up period—40% 182/451). Adherence to guideline recommended medications did not differ significantly between levels of intervention use (*p* = 0.44). At 12 months, the intervention group had a non-significant higher proportion of participants achieving the primary outcome of ≥80% medication days covered than in the control group (32.8% vs. 29.9%; RR 1.07 [95% CI 0.88–1.20]) (Fig. 3). The relative risk was broadly unchanged when adjusted in multivariate analyses for age, sex, and diabetes status. There were no significant differences between the control and intervention groups on the primary outcome for any of our pre-specified sub-groups of gender, age, baseline eHEALS, and CVD subgroups (Fig. 4).

At 12 months, there was a borderline improvement in BP and LDL control rates in intervention vs. control (17.1% vs. 12.1%, RR 1.41 95% CI 0.98–2.03, *p* = 0.07), however control rates remained low overall in both study arms. There were no significant differences between the intervention and control groups in mean LDL cholesterol (2.5 vs. 2.4 mmol/L, mean difference −0.08 mmol/L, 95% CI −0.22 to 0.05, *p* = 0.24) and SBP (136.3 vs. 136.4 mmHg, mean difference 0.12 mmHg, 95% CI −2.21 to 2.45, *p* = 0.92). For lifestyle behaviors, there were significantly more participants meeting recommended levels for physical activity (87 vs 79.7%, *p* = 0.02) in the intervention than the control group (Fig. 3). There were no significant differences in any of other lifestyle-related behaviors including quality of life scores and HLQ scores. For e-health literacy scores there were significant improvements in participants meeting the pre-defined threshold of high e-health literacy in the intervention vs. control arm (72.6% vs. 64.0%, *p* = 0.016). There were few all-cause hospitalizations (59 vs. 54) and deaths (2 vs. 1) in both intervention and control groups, respectively. Owing to small numbers significance testing was not performed.

## Discussion

Among patients with or at high risk of CVD, a consumer-focused and EHR-integrated software application did not improve adherence to guideline recommended medicines. The study population had low to very low medication adherence rates and concomitant risk factor control rates at baseline and there was only a marginal improvement post-intervention. The minimal effects on most outcomes occurred despite reasonable implementation fidelity. The findings are concerning given this population is at high to very high risk of experiencing either a first or subsequent CVD event. The evidence base for guideline-recommended treatments (BP-lowering medications and statins) is well established and when these medications are used in combination they can lower risk of a CVD event by around 40%^15^. Optimal medication use (combined BP and statin medication coverage for at least 80% of the previous 12-month period) was observed in only around one-third of people with around a half of people taking BP medications consistently and only 40% taking a statin over a 12-month period. These gaps are well known and in the Australian primary care context have changed little over the last two decades.

The adherence literature related to CVD medications has repeatedly shown that adherence is heterogeneously impacted by disease factors, therapy factors, healthcare factors, patient factors and, social factors^16^. As such, strategies to improve adherence tend to have mixed success. The large treatment gaps identified in our study and the minimal movement with this intervention suggests more intensive, system wide strategies are needed to address this intractable problem. Traditionally, intervention approaches look at supply side (provider and system) strategies and demand side (consumer-focused) strategies. Digital health interventions for cardiovascular risk are proliferating and effect sizes vary greatly. On the consumer side, the Text2PreventCVD Collaboration found text messaging systems have modest but potentially important reductions in cardiovascular risk factors^17^. Similarly, supply-side interventions to improve quality include audit and feedback, decision support tend to show mixed outcomes^18^. Patient and provider education strategies are moderately successful. A recent systematic review of strategies to increase statin prescribing rates shed some insights on both sides—patient education initiatives were effective in four of seven trials and two trials that combines electronic decision support with audit and feedback were effective^19^. More recently, behavioral economics studies are emerging but also inconclusive to date—one recent study used payments to providers and/or patients to improve adherence rates to statins and found that only the combined provider and patient incentives were effective in lowering LDL cholesterol and that overall the intervention effects were modest and not cost-effective^20^.

This mixed evidence base suggests that contextual factors at multiple levels—health system, service, provider, patient, and community levels—play a role in influencing the effectiveness of these strategies. The recently published Non-adoption, Abandonment, Scale-up, Spread and Sustainability (NASS) framework provides a mechanism for explicitly assessing complexity across multiple domains to understand adoption barriers and enablers with technology interventions^21^. Two NASS domains of particular importance in this study was the value proposition to users and the adopter system. The CONNECT intervention has multidimensional components and although it appeared to be viewed favorably, particularly for goal setting and taking lifestyle actions it may have had little value to users in relation to medication management. There was also complexity with the adopter system which was attempting to promote a more engaged discussion between provider and patient by integrating the application with EHR systems. This link was perhaps not sufficiently strong and research on the impact of direct messaging between patient and providers is an area for greater exploration. A more detailed examination of the impact on health-related behavior and how the EHR-linked strategy was received, used, and accepted by patients and providers in this study has been reported elsewhere^19^.

Importantly, in this study there was some misalignment in results in terms of medication prescription and risk factor measurements and qualitative consumer/patient usefulness and perceived value. This is a common potential problem for RCTs that have a focus on behavior change based on complex interventions where there are multiple moving parts^22^. Together with the improvements in self-reported physical activity, our findings suggest there may have been some value to users for lifestyle changes and motivation. For example, qualitative research conducted alongside this RCT found that 40% of participants reported using the web-app improved their mental health and well-being, 47% reported higher physical activity levels and 61% reported healthier eating^23^. In addition, the qualitative research found 73% of users reported benefiting from personalized CVD risk score; 69% liked the goal tracking; 52% benefited from the risk factor self-monitoring and 54% liked the motivational health tips^24^. The observed disparity between objective clinical outcomes and patient preferences is an important consideration when evaluating this research and future RCTs of complex interventions. Other studies have also highlighted the importance of relevance of outcome measures to consumers/patients^25^. This is an area that requires further research to help understand how future studies can ensure emphasis on outcomes that are of high value to patients but are also scientifically robust so we can most effectively estimate the potential benefits of digital health interventions that are consumer-directed.

Study limitations include the following. First, as mentioned in the “Methods” section, the study was originally powered on risk factor control and we were aiming to recruit 2000 individuals. This resulted in a slight imbalance in numbers in the control and intervention groups although no major difference in measures. Despite low withdrawal rates, recruitment proved challenging where primary care practices are not well supported to undertake research. We had to revise the recruitment target to 1000 patients and a more appropriate primary outcome (prescription of evidence-based medications). It is possible that given the trend to significance in risk factor target control that the study was underpowered to show an effect, however, even if such an effect was observed it would have been modest at best and the broad conclusions remain unchanged. Second, there was a much higher proportion of men recruited to the study than women. The reasons for this are complex and are related to both a higher proportion of men identified at high CVD risk, but also a higher proportion of men than women agreeing to participate in the study. This is important given the emerging data on gender disparities in both health status but also health care. Third, the study was conducted in mainly urban primary care practices in one city and practice level factors may be different in other settings which may lead to different conclusions. Also, two practices experienced challenges with installing the software to upload data to the shared EHR and this limited the ability of these sites to refresh information from the patient record into the CONNECT application. Finally, due to the low numbers of ACCHSs recruited, we are not able to make any scientific conclusions about differential impacts for Aboriginal and Torres Strait Islander people compared with the general study population and hence have not attempted to do so. This would need to be the subject of further specialized research.

In conclusion, a consumer app integrated with primary health care EHRs was not effective in increasing medication usage in a population at high risk of CVD events with low pre-existing use of recommended medications. Borderline improvements in risk factor control and modest behavioral changes were observed. When considering the current evidence of behavior change strategies for CVD risk reduction, this study affirms that such interventions remain challenging to implement and to achieve clinical effectiveness. Innovative approaches to intensify the effects of such interventions are needed and it is likely such approaches need to target multiple levels of the health system.

## Methods

### Study design and participants

The Consumer Navigation of Electronic Cardiovascular Tools (CONNECT) study was a parallel-design, single-blind randomized clinical trial enrolling 934 patients with, or at high risk of, CVD presenting at 23 Australian primary care practices and one Aboriginal Community Controlled Health Service (ACCHS) with an average follow-up of 12 months (Fig. 1). The protocol is detailed elsewhere^26^. Participants in both intervention and control groups received usual health care, but those in the intervention arm were given access to a web application that was integrated with their primary health care EHR. Participants provided written informed consent. Ethical approval was obtained from the University of Sydney Human Research Ethics Committee (2013/716) and the New South Wales Aboriginal Health and Medical Research Council (959/13).

Consenting adult patients (>18 years) with access to the internet at least once a month via mobile phone, tablet or computer, and at moderate to high risk of a CVD event were eligible to participate. Participants had to have presented to a participating primary care practice or health service twice in the last 2 years and once in the last 6 months. Moderate to high cardiovascular risk was defined as having (i) a 5-year CVD risk ≥10% using the Framingham risk equation^27^; (ii) a clinically high risk condition based on Australian guidelines (Aboriginal/Torres Strait Islander and age >75 years, diabetes and age>60 years, diabetes and albuminuria, eGFR<45 ml/min, systolic blood pressure (BP) ≥ 180 mmHg, diastolic BP ≥ 110 mmHg, total cholesterol >7.5 mmol/L) or an established CVD diagnosis (ischemic heart disease, stroke/transient ischemic attack, peripheral vascular disease)^27^. Potential participants with a severe intellectual disability, or insufficient English to provide written, informed consent were excluded.

### Recruitment

Primary health care services in Sydney, New South Wales, Australia were recruited. Of these, 23 were general practices and one was an ACCHS. Software to enable integration of the EHR with the consumer portal was installed at each participating site. A reimbursement of AUD$50 per participant recruited was made to participating practices to support administrative time of practice staff. All software license costs and technical support were provided free of charge to the study sites for the duration of the trial. Royal Australian College of General Practitioners (GPs) Quality Improvement and Continuing Professional Development points were also offered to participating GPs to support their professional development requirements in terms of contributing to research and quality improvement.

Recruitment took place between November 2014 and May 2017 (follow-up until July 2018). Potential participants who met attendance and clinical eligibility criteria were initially identified by study personnel using a data extraction tool routinely used in Australian primary health care software systems. Once identified, the list of potential participants was reviewed by the attending GP to identify unsuitable patients. All others were then mailed a study invitation letter from their GP and received a follow-up telephone call from study personnel. During the phone call, eligibility including internet access were confirmed. If the person was interested in participating, an in-person appointment at the practice or health service was arranged during which written informed consent was obtained prior to baseline assessment and randomization. Consent was separately obtained for linkage with federal administrative data from the Australian Medicare Benefits Scheme (MBS), to determine health service utilization and, the Pharmaceutical Benefits Scheme (PBS), which contains the dispensing data required to ascertain proportion of days covered with guideline recommended medications.

### Randomization and masking

Participants were randomized to either have access to the CONNECT web application in addition to their usual health care (intervention) or receive their usual health care without access to the web-application (control). In both groups, any advice and/or other interventions provided by the GP/health service continued at their discretion. Randomization was conducted independently using a central computer-based randomization service with a 1:1 ratio. A permuted block sequence was used with stratification by practice, baseline CVD risk status and, Aboriginal/Torres Strait Islander status. The random allocation sequence was concealed from study personnel, and took place after collection of baseline data. Study personnel taking baseline and follow-up measurements were blinded to group allocation and participants were asked not to discuss whether they were receiving the intervention or not during their follow-up visit.

### Intervention

The CONNECT digital health intervention was a consumer-focused, responsive web application with integration of data from the primary health care EHR. It was accessible on any internet-enabled device (smartphone, tablet, laptop, or personal computer) and was developed using a persuasive and user-centered design process^28^. Prior to participant recruitment, software was installed at each participating primary care service to enable upload of selected personal health data into the patients’ secure portal (Extensia Pty Ltd, Brisbane, Australia). Uploaded data included medical diagnoses, prescribed medications, physical measurements (weight, waist circumference and, blood pressure), cholesterol record and hemoglobin A1c (HbA1c) for diabetic patients. The consumer application has multiple components (Fig. 2) to encourage participants to: (i) use every-day familiar devices to increase understanding of the relationship to CVD prevention of lifestyle-related behavior, medication adherence and, regular discussion of these topics with their GP; and (ii) use one or more of self-monitoring, goal setting and, digital messaging functions to facilitate better adherence to these actions. Registered participants had access to numerous features that facilitated knowledge, support and, goal-setting in relation to their personal cardiovascular risk including:

- An auto-populated list of their current medical conditions and prescribed medications with links to more detailed information to enhance knowledge.
- A personalized CVD risk score where patients could see the relationship of their risk factors to the score estimation, then use interactive functionality to visually see the impact of managing their risk factors on their absolute risk (Fig. 2).
- Interactive tools and resources to assist with care navigation; alongside data imported from their EHR where patients could log additional physical measurements taken at home and track their progress with, for example, blood pressure control or weight reduction if relevant. Calendar links also enabled the patient to record due dates for test updates, for example cholesterol measurement.
- Interactive goal-setting based on healthier eating, physical activity, smoking cessation and emotional well-being as well as goal achievement tracking with virtual rewards to facilitate and motivate lifestyle changes.
- An interactive social media component with which participants could read and/or write comments, ask questions or share stories that was moderated by trained clinical staff.
- Optional receipt of personalized CVD prevention tips and motivational messages related to diet, medications and lifestyle via email and/or short message service (SMS) that were developed using a published process^29^ and have previously been found to be effective^11^ and useful for patients^30^ in improving cardiovascular risk.

Study personnel supported intervention arm participants over 12 months using standard protocols to ensure uniformity of support activities and included health professionals with nursing, dietetics, and pharmacy training. Participants were trained in use of the application either in person or by telephone and provided with a printed reference guide if needed. Thereafter, they were contacted by telephone and/or email at scheduled intervals: week 2, week 6, week 12, and week 26. During these routine support calls, staff answered questions, repeated aspects of the initial training if requested, explained clinical content if needed, and addressed navigation, function, or other software-related issues. All communications were logged by time requirement and content, and software trouble-shooting was referred to a technical help desk. Participants could contact research staff by telephone or email whenever they needed additional support. To ensure blinding of outcome assessments, different personnel supported the intervention participants to those who conducted the baseline and 12-month assessments.

### Data collection procedures

Primary data were collected at face-to-face assessments at baseline and face-to-face or telephone assessments at end of study (12 months) by research assistants who were blinded to group allocation. A Standard Operating Procedure was followed by all research assistants to optimize uniformity and completeness of data collection and to ensure standardization of physical measurements and data entry. Data were entered into a case report form and a purpose-built, secure online database. The software installed at each practice or health service to facilitate integration of the EHR with the consumer portal also enabled relevant clinical data to be extracted during the study period. In addition, PBS and MBS data were obtained from the Australian Government Department of Human Services to assess prescription medications dispensed. Site monitoring visits were performed periodically to ensure quality documentation, correct software function, and adherence to various milestones for study personnel contact in the follow-up period for intervention arm participants.

### Outcomes

The primary outcome was the proportion of days covered with guideline recommended medications at 12 months. This was defined based on the proportion of maximum medication dispensed from the patient’s pharmacy using national PBS administrative dispensing data. All medications of interest for this study are processed via this system regardless of the pharmacy visited. The primary outcome was defined as met if at end of study ≥80% of maximum medication had been dispensed in the previous 12 months for at least one BP-lowering medication AND a statin medication. For people with or at high risk of CVD, Australian guidelines recommend prescription of at least one BP lowering medication and a statin unless contraindicated^27^. People with established CVD are additionally recommended an anti-thrombotic agent (most commonly aspirin) however, because aspirin is usually available over the counter and is not reliably captured in the national PBS dataset we did not include it in the primary outcome.

Secondary and tertiary outcomes at 12 months included:

1. The proportion of participants whose BP AND fasting low-density lipoprotein (LDL) cholesterol were meeting Australian guideline targets (defined as: ≤130/80 mmHg for CVD, diabetes, or albuminuria or ≤140/90 mmHg for all others, AND LDL-cholesterol <2.0 mmol/L)^27^.
2. Proportion meeting individual targets for BP and LDL cholesterol.
3. Mean difference in SBP and LDL levels.
4. Proportion of days covered with BP lowering medication and statin medication separately.
5. Smoking—point abstinence (verified by carbon monoxide meter where CO > 8 ppm represents recent tobacco smoking)^31^.
6. Obesity—proportion with a body mass index >30 kg/m^2^.
7. Self-reported physical activity based on World Health Organization (WHO) Global Physical Activity Questionnaire^32^.
8. Health-related Quality of life—EQ5D (version 5L with Australian standardized weights)^33^.
9. Fruit and vegetable intake, fish, salt, and saturated fat intake—self-reported portions consumed in 7 days prior and compared with published guidelines recommendations^34^.
10. Health Literacy (Health Literacy Questionnaire, HLQ)^24^.
11. e-health literacy (eHealth literacy score, eHEALS) with a threshold score of 26 set as an estimate of high or low eHealth literacy where higher scores represent better eHealth literacy^35^.
12. All-cause mortality (medical records); cardiovascular and renal events, new onset diabetes (self-report verified by the primary care record) and; hospital admissions (self-report verified by primary care record).

In our original study protocol the primary outcome was BP and LDL target attainment (secondary outcome number 1 listed above), however due to our inability to reach the original recruitment target of 2000 participants, the study steering committee and ethics committee approved changing this to a secondary outcome and making medication adherence our primary outcome. This was implemented before end of study data collection commenced.

### Statistical analyses

Using the pre-randomization baseline rates, we assumed the proportion of people with >80% coverage with guideline-recommended medications was 28%. A total sample size of 1000 participants, allowing for a 20% loss to follow-up would have 90% power to detect an absolute improvement of at least 10% using two-sided tests, with *p* values of <0.05 judged as significant. For the original primary outcome of BP and LDL target attainment, this sample size provided 80% power to detect a 7% absolute improvement, assuming a baseline control rate of 11%. All statistical analyses were conducted blinded to group allocation.

A pre-specified statistical analysis plan that was finalized prior to database lock was followed. The analysis was done by an independent statistician using SAS (version 9.3). Primary analyses were unadjusted, following an intention-to-treat principle and conducted blind to treatment allocation. Multivariate analyses were performed to adjust for any significant differences between each study arm. Pre-specified sub-group analyses were conducted to compare outcomes based on gender, age, baseline, eHEALS, and CVD status (established CVD compared vs. high CVD risk). Mean risk factor levels were compared between groups in terms of relative risks (RR), 95% confidence intervals (CIs), and two-sided *p* values. Characteristics were compared between groups using independent *t* tests for continuous or *Χ*^2^ tests for categorical variables. Mann–Whitney *U* tests were used where data were not normally distributed.

### Reporting summary

Further information on research design is available in the Nature Research Reporting Summary linked to this article.

## Supplementary information

Reporting Summary

## Data Availability

The data that support the findings of this study are available from the corresponding author upon reasonable request.

## Code availability

The code used to derive the findings of this study are available from the corresponding author upon reasonable request. Analysis was conducted by an independent statistician using SAS (version 9.3).
